# Victims of War: Dehydroepiandrosterone Concentrations in Hair and Their Associations with Trauma Sequelae in Palestinian Adolescents Living in the West Bank

**DOI:** 10.3390/brainsci9020020

**Published:** 2019-01-23

**Authors:** Lena Schindler, Mohammed Shaheen, Rotem Saar-Ashkenazy, Kifah Bani Odeh, Sophia-Helen Sass, Alon Friedman, Clemens Kirschbaum

**Affiliations:** 1 Faculty of Psychology, Technische Universität Dresden, 01069 Dresden, Germany; sophia-helen.sass@mailbox.tu-dresden.de (S.-H.S.); clemens.kirschbaum@tu-dresden.de (C.K.); 2 Faculty of Public Health, Al-Quds University, P.O. Box 4006, 90612 Abu Dees, West Bank, Palestine; kifah_odeh@hotmail.com; 3 Department of Cognitive Neuroscience and Zlotowski Center for Neuroscience, Ben-Gurion University of the Negev, P.O. Box 653, 8410501 Beer-Sheva, Israel; saar.rotem@gmail.com (R.S.-A.); alonf@bgu.ac.il (A.F.); 4 Faculty of Social Work, Ashkelon Academic College, 7821100 Ashkelon, Israel; 5 Department of Medical Neuroscience, Faculty of Medicine, Dalhousie University, P.O. Box 15000, Halifax, NS B3H 4R2, Canada

**Keywords:** DHEA, cortisol, hair, traumatic stress, posttraumatic stress disorder, violent conflict, coping

## Abstract

Due to its anti-glucocorticoid properties, the steroid hormone dehydroepiandrosterone (DHEA) might play a role for coping with traumatic stress and posttraumatic stress disorder (PTSD). The majority of studies report elevated DHEA secretion and decreased cortisol/DHEA ratio associated with traumatic stress, however, contrasting results have also been published. One reason for this heterogeneity might be that in past studies, DHEA has been measured in plasma or saliva samples reflecting acute hormone levels. In comparison, the current study assessed the hair levels of DHEA and cortisol as long-term markers along with self-reported data on psychopathology and coping in 92 female adolescents aged 11–16 from the West Bank affected by the Israeli–Palestinian conflict. Results showed that trauma-exposed individuals had significantly higher DHEA levels (*p* = 0.013) and lower cortisol/DHEA ratios (*p* = 0.036) than participants from the non-trauma group. Furthermore, DHEA and cortisol/DHEA ratio emerged as associated with trauma load and timing, but not with coping. By applying the novel method of DHEA analysis from hair samples, this study adds to the growing literature on the interplay of DHEA, cortisol, traumatic stress and coping, and provides valuable starting points for further research.

## 1. Introduction

Ongoing traumatic stress, as caused by long-lasting political instabilities and armed conflicts, is considered a severe psychological burden, frequently resulting in the development of posttraumatic stress disorder (PTSD). This psychological condition, characterized by symptoms of intrusion, avoidance, and alterations in cognition, mood and arousal [1] is accompanied by typical changes of the physiological stress system, i.e., the hypothalamic–pituitary–adrenal (HPA) axis [2], with childhood and adolescence considered as particularly vulnerable phases [3]. Such changes are fairly well-documented for the central HPA axis hormone cortisol in multiple studies examining blood, urine, saliva or hair samples [4]. The prevailing picture is an up-regulation during or in the immediate aftermath of traumatic stress [5,6,7,8] and a consequent down-regulation more distal to exposure [9,10]. Together, these findings of hyper-, followed by hyposecretion as a function of timing, frequency, and severity of traumatic stress can be subsumed in an encompassing building-block model of traumatic stress [4]. However, for other biomarkers associated with the HPA axis, the results are much sparser. One marker that has been gaining increasing attention in traumatic stress research is the steroid hormone dehydroepiandrosterone (DHEA). DHEA is synthesized in the central nervous system (CNS), but also in the adrenal cortex and the gonads together with its metabolite DHEA-S [11,12]. Similar to cortisol, DHEA is secreted following adrenocorticotropic hormone (ACTH) release, shows a circadian rhythm and is influenced by gender and age [11,13]. Currently, there still is much debate on the exact properties of DHEA [11,14]. There seems to be a dose-dependent relationship, with too low, but also too high levels of DHEA being discussed as ineffective, or even harmful [11]. Of note, abundant insights from animal, but also human studies suggest that DHEA and cortisol serve interconnected, but largely antagonistic functions, e.g., in the shape of the neuro-protective properties of DHEA counteracting the neurotoxic effects of prolonged cortisol secretion, or by opposing effects on neuro-genesis, immunological processes, or memory [14]. Consequently, the maintenance of an appropriate balance between cortisol and DHEA seems to be highly important for homeostasis [14]. However, on a psychological base, it is still largely unclear whether DHEA is a mere marker of stress, or rather a direct correlate of psychological coping and resilience [11,15].

A meta-analytic review [12] reported higher basal DHEA levels in traumatized individuals with and without PTSD compared to non-traumatized controls, suggesting an effect of traumatization per se. Overall, results proved to be heterogeneous, with some researchers reporting higher [16,17,18,19] and others lower [20,21] or unchanged baseline DHEA concentrations in PTSD [22,23,24,25,26,27,28,29,30]. The picture is equally mixed regarding associations of DHEA with PTSD symptomatology, with some studies reporting positive [17], and others no associations [16,18,24,27,28,29,30,31]. Notably, there is also some support for the attributed protective effect of DHEA [11] in PTSD. One study found increases of DHEA over psychotherapy in therapy responders, while for non-responders, the levels decreased [32], and another reported positive associations of DHEA and symptoms improvement over psychotherapy [16]. Furthermore, there are studies reporting an inverse relationship between DHEA and resilience/coping [16,33]. 

Due to the debated antagonistic effects of cortisol and DHEA, some researchers argue that the ratio of both markers, rather than one of the markers alone, might have become dysregulated in PTSD [11]. Support stems from studies that report lower cortisol/DHEA ratio in traumatization and/or PTSD [16,17,24,29,31]. Yet other studies found no differences at baseline with unstimulated ratios [19,20,23,34]. Altogether, the majority of studies report inverse associations of cortisol/DHEA ratio and symptomatology [16,17], while others report null-results [24,29,31]. Furthermore, studies on recovery find lower pre-treatment cortisol/DHEA ratios to predict higher symptom improvement [35].

In summary, data regarding DHEA, cortisol/DHEA, traumatic stress, and coping still seems to be very inconclusive. While this might stem from a non-linear dose- and time-dependent impact of traumatization and/or PTSD on the HPA axis [4], it might also be attributable to differences in study designs, e.g., the state of traumatization in the control groups, the small sample sizes, or the mode of biomarker sampling applied. Notably, available data so far almost exclusively stems from saliva [17,23,33] and blood samples [16,18,19,20,21,22,24,25,26,27,29,31,32,34,35]. While those methods are well-suited for capturing short-term alterations in hormonal levels, e.g., as a consequence of acute stress, they are less ideal for the assessment of chronic conditions due to their high sensitivity to situational influences, the underlying diurnal cycle of endocrine levels and the short time spans (minutes to hours) of secretion covered [36]. A novel method to assess long-term steroid secretion is the analysis of hair samples [37], enabling researchers to non-invasively gain insights into the steroid secretion of the previous weeks to months based on a hair growth rate of 1 cm per month [38]. While this method already is fairly well-established for cortisol [4,36], the assessment of hair DHEA can be considered innovative in trauma research. Furthermore, while it is widely accepted that childhood and adolescence mark particularly sensitive phases for the effects of traumatic stress on physiological processes [39], studies on juvenile participants are sparse in DHEA research. 

In this study, we sought to examine hair DHEA levels in the context of chronic traumatic stress and coping in adolescents living in the West Bank. We expected, in line with available meta-analytic and review data [4,12], increased hair DHEA and cortisol levels and decreased cortisol/DHEA ratio in individuals with trauma history compared to non-exposed adolescents, with adolescents with a preliminary PTSD diagnosis showing the largest alterations. Furthermore, we expected associations of hair DHEA, cortisol and cortisol/DHEA ratio with trauma load and clinical symptomatology. In a previous study [5], we could demonstrate elevated hair cortisol concentrations in particular in participants with PTSD and low levels of the resilience factor “Sense of Coherence” (SoC), described as a global mindset to interpret the world as comprehensible, manageable and meaningful, which may be assessed on an individual and on a family level [40]. This is in accordance with several studies suggesting the important role SoC might play for psychological well-being in the aftermath of severe and/or traumatic stress on a self-report [41,42,43,44], but also on a psychoendocrine level [45,46]. Consequently, next to cortisol, we estimated SoC and Sense of Family Coherence (SoFC) to also be associated with hair DHEA and cortisol/DHEA ratio, and therefore hoped to contribute to the ongoing debate of the exact properties of DHEA.

## 2. Materials and Methods

The present sample is a subsample of participants from a previously published study [5] for whom hair DHEA data was available. Data from one hundred participants, among them ninety-two female and eight male adolescents, could be obtained. Due to the small number of male participants, attributable to the minimum required hair length of 3 cm, and the great effect of gender on the HPA axis reported in the literature [39], it was decided to exclude male participants from the following analyses. Consequently, the sample consisted of 92 female participants between 11 and 16 years (*M* = 13.49; *SD* = 1.3) assessed in 2016 in the West Bank governorate of Ramallah. Exclusion criteria were, apart from hair length shorter than 3 cm at the posterior vertex region of the scalp, physical diseases of the CNS, traumatic brain injuries, brain tumors, epilepsy, and/or use of and glucocorticoid or CNS-active medications. The recruiting was conducted with the aid of community-based organizations, who approached eligible adolescents and their parents/legal guardians with detailed study information. After parents/legal guardians provided informed consent, the adolescents were invited to local communities in small groups of 8–10 participants. Participants were instructed to complete questionnaires regarding sociodemographic (age, gender, number of siblings, self-assessment of physical, academic and family income level as low, medium or high), health-related (physical diseases, medication intake, smoking and drinking habits, drug use), and clinical data and had hair samples taken. 

**Figure 1:**
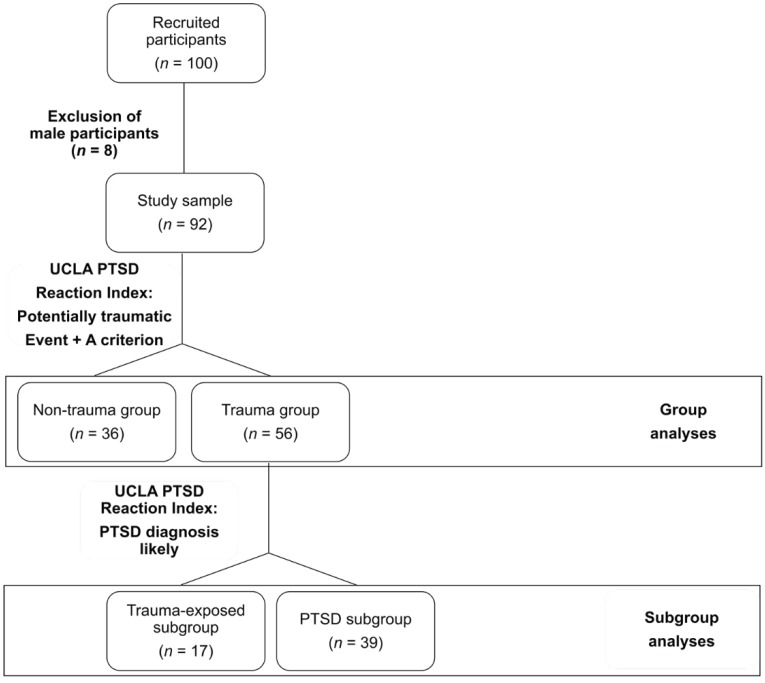
Flowchart of group allocation based on the UCLA PTSD Reaction Index for DSM IV, Child Version.

Participants reported to have experienced or witnessed a plethora of different traumatic experiences, namely, accidents (*n* = 45), acts of war (*n* = 40), physical assaults experienced at home (*n* = 28) or in other places of daily life (*n* = 34), sexual assaults (*n* = 6), witnessing somebody die (*n* = 24), death of a close person (*n* = 34), illness (*n* = 19), or other experiences (*n* = 17). Participants were divided into groups based on the UCLA PTSD Reaction Index for DSM-IV, Child Version [47] (see Figure 1). If they had reported at least one potentially traumatic event and fulfilled the A criterion of the DSM-IV [48], they were allocated to the trauma group (*n* = 56), if not, they were considered part of the non-trauma group (*n* = 36). The trauma group was further divided into subgroups based on preliminary PTSD symptomatology in trauma-exposed with PTSD unlikely (trauma-exposed subgroup, *n* = 17) and trauma-exposed with PTSD likely (PTSD subgroup, *n* = 39) individuals. The study was conducted in accordance with the latest version of the declaration of Helsinki and was approved by the local Ethics Committee of the Al-Quds University. 

Information about demographic and hair-related data was collected using self-developed checklists. Trauma exposure and PTSD symptomatology were assessed via the UCLA PTSD Reaction Index for DSM IV, Child Version [47]. While this scale is not intended to establish a clinical diagnosis, it allows the principal classification of a likely full (A criterion and intrusion, avoidance and arousal symptoms met) or partial (A criterion and two of three symptom clusters met) clinical picture of PTSD with high sensitivity and specificity [49]. Trait anxiety was examined using the trait scale of the State-Trait Anxiety Scale [50]. The Center for Epidemiological Studies Depression Scale [51] was used to assess the presence and severity of depressive symptomatology. For an examination of the resilience factor SoC, the 13-item version of the SoC Scale [40] and the SoFC Scale [52] were applied. Lastly, for an assessment of sleeping problems, the Mini Sleep Questionnaire [53] was chosen. All questionnaires had been translated to Arabic by a professional translator.

Hair samples were cut as close as possible to the scalp at the posterior vertex region [36], the region with the proposedly most uniform hair growth rate [54]. Two to three hair samples with an approximate overall diameter of 3 mm were sampled, wrapped in aluminum foil, stored in a dry, dark place, and sent to the biopsychological laboratory of the Technische Universität Dresden. The 3 cm hair segment most proximal to the scalp, reflecting three months of hormone secretion based on a growth rate of 1 cm per month [38] was analyzed following the published liquid chromatography tandem mass spectrometry (LC-MS/MS) protocol for cortisol and DHEA [37]. In brief, hair samples were washed for 2 × 3 min in 3 mL isopropanol. For glucocorticoid extraction, 7.5 mg of non-pulverized hair were incubated with 1.8 mL of methanol for 18 hours at room temperature. Then, the methanol was evaporated at 50 °C under a constant stream of nitrogen until the samples were completely dried. As a last step, the dry residue was resuspended using 225 μL double-distilled water, from which 100 μL were used for LC-MS/MS procedure. Sensitivity, specificity and reliability of this method has been shown, with inter- and intra-assay coefficients of variance ranging between 4.5% and 9.1% for DHEA and between 3.7% and 8.8% for cortisol [37]. 

All statistical analyses were done via SPSS for Windows, version 25 [55], for analyses, and R [56], for figures. As hair DHEA and cortisol data was positively skewed, log-transformed values were used for the analyses, while for descriptive purposes, original units (pg/mg) are reported in figures and tables. For eight participants (8.7%), DHEA hair levels were lower than the detection limit, which was dealt with by conservative ad-hoc single imputation (replacing the relevant non-detectable values with detection limit/2 such as recommended for <15% of non-detectables) [57]. Due to high outlying values of more than three SD above the mean, one participant from the PTSD subgroup was excluded from hair DHEA analyses and one participant from the non-trauma group was excluded from hair cortisol analyses. Group comparisons regarding demographic, hair-related and clinical data were conducted using analyses of variance (ANOVAs) with Bonferroni-corrected post-hoc tests for continuous and χ² contingency tables for dichotomous variables, respectively. Generalized linear models (GLM) were chosen to analyze hair cortisol, DHEA and cortisol/DHEA ratio differences between non-trauma, trauma and PTSD participants. Age and, due to the detected differences on group level, depressive symptomatology (*CES-DC*) and the severity of sleeping problems (*MSQ*) were added as covariates. However, this did not have any significant influences on the models (all *ps* ≥ 0.273). Correlation analyses were done using Bravais-Pearson (r) or Spearman (r_sp_) correlations for continuous or ordinal data.

## 3. Results

### 3.1. Psychological Measures

Groups proved to be well-matched regarding sociodemographic and health-related variables (all *ps* ≥ 0.077 see Table 1). However, significant group differences regarding the clinical variables emerged (see Table 2), with participants from both the trauma-exposed and the PTSD subgroup reporting to have experienced more potentially traumatic events (UCLA PTSD Reaction Index) than the non-trauma group (*p* < 0.001), albeit without significant group differences regarding the time of the worst potentially traumatic event. Furthermore, PTSD participants proved to have the highest levels of arousal, overall PTSD severity and sleeping difficulties (MSQ) (all *ps* ≤ 0.045), as well as the highest proportion of dissociations during potentially traumatic events (*p* < 0.001). Notably, regarding intrusion severity, group differences only emerged at the trend level, while for avoidance, both the PTSD and the non-trauma participants scored higher than the trauma-exposed subgroup (all *ps* ≤ 0.03). 

Among the PTSD subgroup, most of the individuals reached the cutoff score for depression (CES-DC), however, the lowest score appeared in the trauma-exposed subgroup who had not developed PTSD. Consequently, the only group difference that emerged regarding the severity of depression symptoms was between the trauma-exposed and PTSD subgroups (*p* = 0.045). Groups did not significantly differ regarding trait anxiety (STAI-Y-Trait), SoC, or SoFC (all *ps* ≥ 0.178). However, it emerged that SoC was inversely associated with the time since the worst traumatic event (r_sp_ = −0.26, *p* = 0.03), as well as with the severity of intrusion (UCLA PTSD Reaction Index, r = −0.26, *p* = 0.012) depressive symptomatology (CES-DC, r = −0.44, *p* < 0.001) as well as with the severity of sleeping problems (MSQ, r = −0.28, *p* = 0.008). For the SoFC, also inverse associations were revealed with the depressive symptom severity (CES-DC, r = −0.31, *p* = −0.003) as well as with sleeping problems at trend level (MSQ, r = −0.2, *p* = 0.051). 

### 3.2. Biomarker Data from Hair Samples

Age, subjective physical development, and storage time of hair samples proved to be unrelated to hair DHEA, cortisol, and cortisol/DHEA ratio (all *ps* ≥ 0.195). There was a trend for a positive correlation between DHEA and cortisol concentrations in hair (*r* = 0.18, *p* = 0.098). 

**Figure 2:**
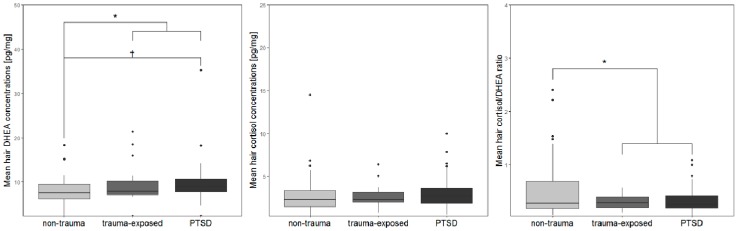
Boxplots of (**A**) DHEA, (**B**) cortisol, and (**C**) cortisol/DHEA ratios from hair samples of the non-trauma, the trauma-exposed and the PTSD participants based on the UCLA PTSD Reaction Index for DSM IV, Child Version.

The GLM of hair DHEA concentrations and trauma status revealed a significant group difference (*F*_1, 89_ = 6.42, *p* = 0.013, η^2^_p_ = 0.067), with higher DHEA levels in trauma-exposed individuals compared to the non-trauma group. When computing subgroup analyses, the effect also emerged (*F*_2, 88_ = 3.2, *p* = 0.046, η^2^_p_ = 0.068), with Bonferroni-corrected post-hoc tests revealing elevated DHEA levels in the PTSD subgroup compared to the non-trauma group at trend level (*p* = 0.055), with trauma-exposed individuals lying in between (see Figure 2). 

For hair cortisol, no significant group differences emerged, neither for trauma status, nor when further dividing the trauma group based on PTSD status (all *ps* ≥ 0.352, see Figure 2). When assessing the cortisol/DHEA ratio, a group effect of trauma status emerged (*F*_1, 90_ = 4.53, *p* = 0.036, η^2^_p_ = 0.048), with lower ratios in the trauma compared to the non-trauma group. A further examination for PTSD status did not lead to statistically significant group differences (*F*_2, 89_ = 2.35, *p* = 0.101, η^2^_p_ = 0.05, see Figure 2). 

However, correlation analyses over the whole sample revealed that hair DHEA was significantly associated with the number of potentially traumatic events (UCLA PTSD Reaction Index, r = 0.29, *p* = 0.006), as well as with sleeping difficulties at trend level (MSQ, r = 0.2, *p* = 0.06). For cortisol and cortisol/DHEA ratio, no significant associations emerged (all *ps* ≥ 0.107). When assessing correlations only for the individuals from the trauma group, associations with SoFC emerged with DHEA at trend level and with the cortisol/DHEA ratio (r = 0.24, *p* = 0.072 and r = −0.31, *p* = 0.019, respectively). For cortisol and cortisol/DHEA ratio, the time since the worst traumatic event (UCLA PTSD Reaction Index) emerged as significant (r_sp_ = 0.41, *p* = 0.002) and trend, respectively (r_sp_ = 0.26, *p* = 0.066). When assessing only those participants with a likely PTSD diagnosis, the number of traumatic events (r = 0.42, *p* = 0.008) as well as the SoFC (r = 0.384, *p* = 0.017) emerged as significantly associated with DHEA levels. For cortisol levels, the time since the worst traumatic event emerged as statistically significant (r_sp_ = 0.44, *p* = 0.007), while SoFC was significantly correlated with the cortisol/DHEA ratio (r = −0.35, *p* = 0.028). Notably, no biomarker associations with SoC emerged (all *ps* ≥ 0.163). 

## 4. Discussion

The current study set out to demonstrate the utility of DHEA extracted from hair samples as a biomarker for traumatic stress and PTSD in a sample of adolescents exposed to the ongoing Israeli-Palestinian crisis in the West Bank. We wanted to examine associations of hair DHEA and resilience/coping, and the interplay of hair DHEA and cortisol as adrenocortical markers of chronic stress.

We confirmed our hypothesis of elevated hair DHEA concentrations in trauma-exposed compared to non-exposed individuals. Further analyses revealed that these elevations were particularly evident in participants with a preliminary diagnosis of PTSD, but elevated levels also emerged in trauma-exposed individuals without preliminary PTSD diagnosis [47]. This finding meshes well with the current literature suggesting alterations following traumatization per se [12], while, however, also being at variance with some studies reporting unchanged [22,23,24,25,26,27,28,29,30] or even lower DHEA concentrations [20,21] in traumatization. 

We also found evidence for decreased cortisol/DHEA ratio in the trauma compared to the non-trauma group, however, hair cortisol concentrations were not significantly different between the groups. The finding of lower cortisol/DHEA ratio in traumatization is in line with several previous studies [16,17,24,29,31]. It must be noted, however, that the results for cortisol are contrary to our hypothesis and at variance with the previously published results on Palestinian adolescents in 2018, where we found elevated hair cortisol concentrations in the PTSD compared to the non-trauma group. While it is improbable that the non-significant findings are a mere effect of the smaller sample size, the current study group originated from a different and smaller area of the West Bank and could have therefore been affected to a different extent by (post-)traumatic stress. Additionally, the study samples differed regarding the availability of data for male participants. However, the clinical characteristics of the participants of the current sample corresponded well with the previously assessed sample, making it improbable that the nonsignificant finding for cortisol reported here might be attributable to less severe psychopathology in the current sample.

There are several theoretical frameworks for an interpretation of these findings. First of all, they might be attributable to time- and dose-dependent endocrine changes [4]. While our findings of DHEA elevations in individuals from the trauma group as well as the positive associations between the number of traumatic events and DHEA concentrations mesh well with this interpretation, currently, the literature on longitudinal trajectories of DHEA secretion following traumatization is much too sparse to corroborate such conclusions. Notably, we also found tentative positive associations of the timing of traumatic stress and cortisol as well as cortisol/DHEA ratio. While this is seemingly at variance with the building block model postulating down-regulation of cortisol with increasing distance to the stressor, it might be explained by the particular living conditions of the sample assessed. Due to the constant presence of direct (e.g., acts of war, violent outbursts), and indirect (e.g., poor infrastructure, substandard medical care) sequelae of the Israeli–Palestinian conflict, and the thusly-resulting stress evident in self-reported data, it is possible that a downregulation of the HPA axis has just not yet taken place, and an earlier traumatization is a mere indicator of overall worse living conditions. On a last note, it must be mentioned that information regarding the timing of traumatic stress was assessed solely in a self-reported fashion, which might have been clouded by inexact memories of the participating individuals. 

This leads to a further hypothesis debated in the literature, namely the observation that early life (traumatic) stress seems to “set“ the HPA axis, and thereby also DHEA secretion, in a certain way, thus exerting particularly fundamental effects on the individual development, and the individual reaction to further, traumatic stress [24,39,58]. Some reports suggest that the earlier the traumatic stress takes place, the bigger the alterations [3], which, again, meshes well with the positive associations we found of cortisol and the cortisol/DHEA ratio with the timing of traumatic stress. A recent notable study tracks DHEA secretion following childhood adversity into the next generation, finding DHEA elevations in the hair samples of new mothers, as well as, on a more tentative level, in those of their newborn children [59], proving the substantial impact early life stress might have on DHEA secretion. However, other studies find no particular effects of adverse childhood events in juvenile and adult populations [28,33,34], highlighting the possible relevance of a plethora of further characteristics next to the timing of traumatic stress, such as type, duration and severity of the traumatic event, which urgently need assessment. 

Anti-glucocorticoid effects of DHEA [11], as well as the frequently reported [13,16] weak associations between cortisol and DHEA were also evident in this study, suggesting that under certain circumstances in the aftermath of traumatic stress, cortisol and DHEA may react in a markedly different manner. The most prominent interpretation is that DHEA is a driving force of coping and resilience [11,12,15], with support from studies finding associations of DHEA with therapy success, resilient functioning and coping [16,32,33]. The fact that these studies do find positive, but also negative associations might again point at a time-dependent pattern of DHEA hyper- and hyposecretion. However, the data from our study cannot corroborate these findings. Apart from very tentative associations of DHEA and cortisol/DHEA ratio with SoFC, we could not confirm our hypotheses of endocrine associations with resilience/coping. Notably, however, as in our previous study [5], SoC and SoFC were inversely associated with certain facets of self-reported psychopathology. 

It might, however, also be possible that DHEA secretion follows a completely different, non-curvilinear trajectory after trauma exposure. One further theory suggests that DHEA levels might be specifically related to acute changes of (post-)traumatic stress [12], e.g., for phases of symptom worsening, but also of symptom improving, like during psychotherapy. DHEA might not indicate mere successful coping, but phases of intense confrontation with traumatic stress in successful or unsuccessful ways. Longitudinal studies closely monitoring the trajectory of DHEA and general endocrine functioning over the course of pathogenesis and therapy are urgently warranted, in order to increase understanding of the psychobiology of traumatic stress.

A major strength of this study is the implementation of hair sampling for the assessment of secretion of DHEA and cortisol of a prolonged period of time (three months). While for studies on cortisol, hair sampling has already become the method of choice when aiming to assess long-term dysregulations [4,36], the assessment of DHEA in hair has not yet received a comparable attention as a potential long-term biomarker of traumatic stress and PTSD. Furthermore, this study is one of very few in DHEA research comparing a PTSD subgroup with both trauma-exposed and non-exposed individuals, supporting the previous findings of DHEA alterations present in traumatization per se [12]. Of note, however, the very specific characteristics of the sample studied must be stressed. Individuals from all three groups live in a highly stressful environment, affected by a constant need for alertness and a prevailing fear of emerging adverse events, like deteriorations of the humanitarian situation or outbreaks of violence. This situation is also mirrored in the prevalence of, in particular, intrusion-like symptoms, avoidance behavior and high arousal in the non-trauma group. The studying of an additional, demographically similar group unaffected by the Israeli-Palestinian conflict would be highly interesting. However, among the Palestinian population, such individuals are almost impossible to find. Of particular relevance, this study is one of very few to assess DHEA levels in adolescents, even though childhood and adolescence are postulated to be a crucial phase for the calibration of the HPA axis [39]. Furthermore, the examination of individuals under ongoing traumatic stress adds new insights into the prevailing debate of the psychophysiological properties of DHEA and its relationship with cortisol. 

The main limitation of the current study is that due to the required hair length of 3 cm, we did not succeed in recruiting enough male participants to guarantee valid statistical analyses. Therefore, we had to constrain our analyses to an all-female sample. While this might have facilitated the interpretation of the DHEA results due to the postulated gender effects on HPA axis functioning coming to effect during puberty [39] it also kept us from examining exactly these gender effects in the context of traumatic stress. Notably, the majority of studies assessing DHEA in the context of traumatic stress solely consist of female samples [12]. Therefore, studies focusing on male participants are urgently warranted. Furthermore, it was not possible for us to directly assess body mass index and pubertal status, confining our study to self-reported evidence regarding those two potential confounders of endocrine functioning [60]. Notably, however, none of the biomarkers assessed proved to be associated to age and physical development, possibly due to the relatively homogenous age range of the participants. Furthermore, apart from the PTSD symptomatology, the levels for depression and trait anxiety were high in the current sample, making it difficult to discern the effects of traumatic stress, depression, and anxiety from one another at an endocrine level. Certain available data suggest an important influence on depression for both cortisol and DHEA secretion [61,62]. In contrast, in our sample, no associations emerged for either depressiveness or anxiety and endocrine secretion. At a conceptual level, we would not necessarily consider those variables as confounders in our sample, but rather as facets completing the clinical picture of individuals living in a high-risk environment and under prolonged and severe traumatic stress. It must further be noted that a high proportion of the participants was wearing a hijab, thereby protecting their hair for the majority of time while outside. While there is first evidence from laboratory studies that the exposure to UV radiation might influence the concentration of endocrine parameters in hair [63,64], this effect has not yet been shown in vivo. Additionally, we do not have reasons to suspect that the subgroups differed regarding the wearing of hijabs. Therefore, this aspect is considered to not undermine the validity of the results. 

## 5. Conclusions

In conclusion: this study is one of the first to implement hair analyses in DHEA research, and yielded evidence for DHEA elevations and decreased cortisol/DHEA ratios in traumatization per se, as well as important associations of endocrine markers with indicators of trauma load and timing, but not with SoC as marker of coping/resilience. These results, albeit needing to be interpreted cautiously due to possible confounding influences, add to the still inconclusive picture of the role of DHEA in traumatic stress. Further, multimodal and longitudinal studies combining different endocrine markers from hair samples are urgently warranted in order to increase the understanding of psychoendocrine mechanisms during and after traumatic stress and the coping with it. 

## Figures and Tables

**Table 1:** Comparison of demographic and hair-related characteristics of study groups (non-trauma, trauma-exposed and PTSD participants) based on the UCLA PTSD Reaction Index for DSM IV, Child Version.

	Non-Trauma Group (*n* = 36)	Trauma-Exposed Subgroup (*n* =17)	PTSD Subgroup (*n* = 39)	Test Statistic	*p*
Age (mean, *SD*)	13.36 (1.29)	13.41 (1.28)	13.64 (1.33)	*F*_2, 89_ = 0.47	0.628
Siblings (mean, *SD*)	5.42 (2.17)	4.18 (1.24)	5.15 (1.76)	*F*_2, 89_ = 2.64	0.077
Physical disease (%)	1 (2.8)	2 (11.8)	2 (5.1)	χ²_2_ = 1.83	0.401
Medication (%)	1 (4.3) ^a^	1 (7.7) ^b^	3 (13) ^c^	χ²_2_ = 1.13	0.567
Smoking (%)	0 (0)	0 (0)	0 (0)	-	-
Alcohol (%)	0 (0)	1 (6.3) ^d^	0 (0)	χ²_2_ = 4.74	0.094
Drugs (%)	0 (0)	0 (0)	0 (0)	-	-
Physical development category	-	-	-	χ²_8_ = 4.16	0.842
Academic level category	-	-	-	χ²_8_ = 5.04	0.753
Family income category	-	-	-	χ²_8_ = 5.16	0.74
Months of hair sample storage (mean, *SD*)	25.54 (0.88)	25.34 (0.79)	25.56 (0.82)	*F*_2, 89_ = 0.46	0.634

^a^ Based on *n* = 23, ^b^ Based on *n* = 13, ^c^ Based on *n* = 23, ^d^ Based on *n* = 16, - = non applicable.

**Table 2:** Clinical and endocrine variables of study groups (non-trauma, trauma-exposed and PTSD participants) based on the UCLA PTSD Reaction Index for DSM IV, Child Version.

	Non-Trauma Group (*n* = 36)	Trauma-Exposed Subgroup (*n* = 17)	PTSD Subgroup (*n* = 39)	Test Statistic	*p*
UCLA PTSD Reaction Index					
Number of potentially traumatic events (mean, *SD*)	1.25 (1.4)	4.35 (2.78)	4.21 (2.52)	*F*_2, 89_ = 20.3	< 0.001^I^
Time since the worst event	-	-	-	χ²_12_ = 5.23	0.95
Dissociation during the event (%)	4 (11.1)	7 (41.2)	22 (56.4)	χ²_2_ = 16.96	< 0.001
Intrusion Severity (mean, *SD*)	5.81 (5.08)	4.88 (4.23)	7.67 (4.07)	*F*_2, 89_ = 2.8	0.066
Avoidance Severity (mean, *SD*)	6.97 (7.44)	2.82 (2.67)	8.92 (3.65)	*F*_2, 89_ = 7.67	0.001 ^II^
Arousal Severity (mean, *SD*)	7.03 (5.94)	6.12 (4.69)	10.59 (3.47)	*F*_2, 89_ = 7.47	0.001 ^III^
PTSD Severity (mean, *SD*)	18.08 (16.85)	12.94 (6.96)	24.44 (8.37)	*F*_2, 89_ = 5.8	0.004 ^IV^
STAI-Y-Trait (mean, *SD*)	45.19 (6.57)	41.47 (6.71)	44.52 (7.21) ^a^	*F*_2, 89_ = 1.76	0.178
CES-DC					
Depression Severity (mean, *SD*)	18.69 (11.02)	16.24 (8.3)	22.64 (6.62)	*F*_2, 89_ = 3.64	0.03 ^V^
Depression Diagnosis (%)	18 (50)	6 (35.3)	34 (87.2)	χ²_2_ = 17.99	< 0.001
MSQ (mean, *SD*)	24.36 (13.68)	27.59 (12.7)	32.74 (11.97)	*F*_2, 89_ = 4.07	0.02 ^VI^
SoC					
SOC-13 (mean, *SD*)	55.56 (10.17)	58.53 (11.77)	54.77 (8.47)	*F*_2, 89_ = 0.88	0.417
SOFC (mean, *SD*)	52.66 (6.86)	53.65 (9.13)	50.69 (6.95)	*F*_2, 89_ = 1.19	0.31
Biomarker					
Hair DHEA (mean, *SD*)	7.77 (3.73)	9.74 (4.77)	9.89 (5.17) ^a^	*F*_2, 88_ = 3.2	0.046 ^c,VII^
Hair cortisol (mean, *SD*)	2.92 (2.56) ^b^	2.71 (1.39)	3.24 (2.02)	*F*_2, 88_ = 0.74	0.48 ^c^
Hair cortisol/DHEA ratio (mean, *SD*)	0.55 (0.59)	0.31 (0.13)	0.36 (0.25)	*F*_2, 89_ = 2.35	0.101 ^c^

UCLA PTSD Reaction Index, UCLA PTSD Reaction Index for DSM-IV, Child Version; STAI-Y-Trait, Trait scale of the State Trait Anxiety Inventory; SOC-13, SoC Scale; SOFC, SoFC Scale; CES-DC, Center for Epidemiological Studies Depression Scale for Children Post-hoc analyses: ^I^ non-trauma < trauma-exposed = PTSD (*ps* < 0.001), ^II^ trauma-exposed < non-trauma = PTSD (*ps* ≤ 0.03), ^III^ non-trauma = trauma-exposed < PTSD (*ps* ≤ 0.006), ^IV^ trauma-exposed < PTSD (*p* = 0.005); non-trauma < PTSD at trend level (*p* = 0.082), ^V^ trauma-exposed < PTSD (*p* = 0.045), ^VI^ non-trauma < PTSD (*p* = 0.017), ^VII^ non-trauma < PTSD at trend level (*p* = 0.055). ^a^ Based on *n* = 38, ^b^ Based on *n* = 35, ^c^ refers to logarithmized data, - = non applicable.
